# Ten years of the Citizen's Electronic Health Record e-SUS Primary Healthcare: in search of an electronic Unified Health System

**DOI:** 10.11606/s1518-8787.2024058005770

**Published:** 2024-06-06

**Authors:** Ianka Cristina Celuppi, Eduarda Talita Bramorski Mohr, Mariano Felisberto, Thiago Serafim Rodrigues, Jades Fernando Hammes, Célio Luiz Cunha, Raul Sidnei Wazlawick, Eduardo Monguilhott Dalmarco

**Affiliations:** I Universidade Federal de Santa Catarina Centro Tecnológico Laboratório Bridge Florianópolis SC Brasil Universidade Federal de Santa Catarina. Centro Tecnológico. Laboratório Bridge. Florianópolis, SC, Brasil; II Universidade Federal de Santa Catarina Centro de Ciências da Saúde Florianópolis SC Brasil Universidade Federal de Santa Catarina. Centro de Ciências da Saúde. Programa de Pós-Graduação em Enfermagem. Florianópolis, SC, Brasil; III Universidade Federal de Santa Catarina Centro de Ciências da Saúde Florianópolis SC Brasil Universidade Federal de Santa Catarina. Centro de Ciências da Saúde. Programa de Pós-Graduação em Farmácia. Florianópolis, SC, Brasil; IV Universidade Federal de Santa Catarina Centro Tecnológico Departamento de Ciências da Computação Florianópolis SC Brasil Universidade Federal de Santa Catarina. Centro Tecnológico. Departamento de Ciências da Computação. Florianópolis, SC, Brasil

**Keywords:** Electronic Health Records, Digital Technology, Primary Healthcare, Health Policy, Unified Health System

## Abstract

**OBJECTIVE:**

Contextualize the adherence to the *Prontuário Eletrônico do Cidadão* (PEC – Citizen's Electronic Health Record) by Brazilian municipalities and the evolution of the electronic strategy of the Unified Health System (e-SUS) for Primary Healthcare (PHC) during its 10 years.

**METHODS:**

This descriptive study added information on adherence to the use of medical records extracted from the database of the *Secretaria de Atenção Primária à Saúde* (SAPS– Primary Healthcare Secretary) of the Federal Government between 2017 and 2022. We analized the number of computerized basic healthcare units that used some electronic medical records, the number of those that used simplified data collection (SDC), and those that implemented the citizen's electronic health record (PEC) in the same period. A descriptive synthesis of the functionalities and modules implemented in the system during its 10 years of development was also carried out.

**RESULTS:**

The adherence of Brazilian municipalities to the PEC has grown exponentially in the last five years, going from 8,930 healthcare units in 2017 to 26,091 in 2022. As expected, while the main functionalities and improvements developed in this decade sought to implement new flows and modules of administrative, clinical care, and care management processes and health service administration, improving aspects of usability and technological infrastructure of the application architecture was also crucial for the success of the system.

**CONCLUSIONS:**

In 2023, the milestone of a decade will be celebrated since the beginning of health records implementation by Brazilian municipalities, marked by technological and infrastructure challenges and improvements and new functionalities that highlight the technological evolution of the e-SUS PHC system and strategy. Despite many other tools, the PEC is arguably Brazil's leading electronic medical record today, as it has always invested in evolution, updating itself in technological and usability opportunities.

## INTRODUCTION

Implementing the Brazilian Unified Health System (SUS) in 1988 enabled the creation of a triad of principles based on the universality, completeness, and equity of health services. In 1990, the *Atenção Primária à Saúde* (APS – Primary Healthcare) was established as a national policy based on Basic Operational Standard 96 (NOB 96), which guaranteed support for the implementation of Family Health and Community Health Agents programs throughout the Brazilian territory^1,2^.

From this perspective, APS plays a central role within the healthcare network organization, being considered the population's primary gateway to the Brazilian health system and expanding healthcare provision in the territory. Currently, according to the 2017 *Política Nacional da Atenção Básica* (PNAB– National Primary Healthcare Policy), APS must encompass integrated care practices and qualified management, valuing multidisciplinary performance in a defined territory^3–5^.

The care provided in APS has significantly reduced the number of hospital admissions over the years, reducing bottlenecks formed mainly by patients with complications arising from chronic diseases, such as hypertension and diabetes^6–8^. However, public management still need to overcome some challenges, such as the recent decrease in vaccination coverage, expanding access to this service, improving the quality of care for chronic conditions, and reducing the transmission of infectious diseases, among other aspects^7,9^.

In the last 10 years, with the advent of technology and the construction of digital ecosystems in the health sector, changes have been incorporated into APS related to computerizing services and adherence to the use of technologies. The e-SUS *Atenção Básica* (e-SUS AB – e-SUS Basic Healthcare) strategy was created following the *Sistema de Informação em Saúde para a Atenção Básica* (SISAB– Health Information System for Basic Healthcare) restructuring in 2013 and aims to expand, restructure, and ensure effective interaction of information systems that permeate health in Brazil^10^. Thus, the e-SUS AB strategy, currently called the e-SUS APS strategy, seeks to expand computerization in the SUS, contributing to the qualification of records, the integration of information, and the optimization of health data analysis through introducing new technologies^10,11^.

In this logic, the Ministry of Health made the *Prontuário Eletrônico do Cidadão* (PEC– Citizen's Electronic Health Record) available for free use by municipalities to facilitate the computerization process of basic healthcare units (BHU) throughout the Brazilian territory. During the 10 years of implementation of the health record, the National Data Centralizer and several mobile applications to support professionals were also developed, such as *e-SUS Atenção Domiciliar* (e-SUS Home Care), *e-SUS Atividade Coletiva* (e-SUS Collective Activity), *e-SUS Território* (e-SUS Territory), *e-SUS Vacinação* (e-SUS Vaccination), and *Gestão e-SUS APS* (e-SUS APS Management)^12^. The e-SUS APS strategy also provides the Simplified Data Collection (SDC) system, considered a transition tool to the computerized model, in which data are collected through forms with subsequent digitization, allowing them to be used in low technology and connectivity infrastructure scenarios^11,13^.

The implementation process of the PEC e-SUS APS can be influenced by the characteristics of the municipalities, such as their location, population density, urbanization, preparation and assistance from municipal management, level of computerization, and technological infrastructure, among others^10^. When associated with other challenges faced by health professionals and managers, such aspects can determine the region's adherence to the project. The literature highlights difficulties in implementing and using the system, such as insufficient material resources in municipalities, lack of training for professionals to use technology, and low internet connectivity^14–16^.

In the meantime, implementing PEC e-SUS APS and other applications focused on the health sector represent structural changes in technologies and work processes already consolidated in Brazil, leading to computerization and the strengthening of digital health in the country. This fact justifies the relevance of developing this study, which summarizes the technological evolution of a successful government policy with a significant impact on Brazilian APS, which completes a decade of implementation in 2023. Given this, this work aims to contextualize the adherence to the Citizen's Electronic Health Record by Brazilian municipalities and the evolution of the e-SUS APS strategy during its 10 years of existence.

## METHODS

This descriptive study added information on adherence to using the PEC, extracted from the *Secretaria de Atenção Primária à Saúde* (SAPS – Primary Healthcare Secretariat) database, between 2017 and 2022. The study also analyzed the functionalities and modules implemented in the system during its 10 years of evolution, presenting an overview of its technological consolidation.

### Adherence of Brazilian municipalities to the PEC e-SUS APS

Over the years, we can observe the growth in PEC's consolidation throughout the Brazilian territory, together with the increase and modernization of BHU. This growth demonstrates a joint effort to unify and defragment the information and services provided in primary healthcare^17,18^. According to information collected from the SAPS database, between 2017 (the year when verification by SAPS began) and 2022, there was an exponential increase in the implementation of the PEC in all Brazilian regions, with emphasis on the Northeast (367.72%), North (256.10%), and Southeast (157.04%) (Table).

**Table:** Basic healthcare units (BHU) with the implementation of the *Prontuário Eletrônico do Cidadão* (PEC- Citizen's Electronic Medical Record), from 2017 to 2022:

Year	Regions of Brazil									
	North		Northeast		Midwest		Southeast		South	
	BHU^a^	PEC^b^	BHU^a^	PEC^b^	BHU^a^	PEC^b^	BHU^a^	PEC^b^	BHU^a^	PEC^b^
2017	751	631 (84.02%)	3,955	2,618 (66.19%)	2,068	1,014 (49.03%)	6,411	2,724 (42.48%)	5,325	1,943 (36.48%)
2018	1,008	841 (83.43%)	4,763	3,232 (67.85%)	2,252	1,255 (55.72%)	7,411	3,273 (44.16%)	5,707	1,997 (34.99%)
2019	1,320	1,099 (83.25%)	6,911	5,018 (72.60%)	2,524	1,618 (64.10%)	9,057	4,708 (51.98%)	6,096	2,354 (38.61%)
2020	1,829	1,553 (84.90%)	9,919	7,905 (79.69%)	2,782	1,891 (67.97%)	11,026	6,364 (57.71%)	6,322	2,557 (40.44%)
2021	2,117	1,802 (85.12%)	11,772	9,843 (83.61%)	2,923	2,090 (71.50%)	11,630	6,705 (57.65%)	6,468	2,495 (38.57%)
2022	2,531	2,247 (88.77%)	13,942	12,245 (87.82%)	2,977	2,201 (73.93%)	12,284	7,002 (57.00%)	6,484	2,396 (36.95%)

Implementation and use of the PEC in Brazil over six years (2017-2022), where ^a^ represents the number of BHU in operation that used some PE in December of each year, and ^b^ represents the number of BHU that chose to implement and use the PEC in December of each year. The percentage (%) represents the number of BHU with PEC^b^ concerning the total BHU^a^. Ministry of Health, 2023 (data collected on 10/17/2023)^19^.

While the total number of BHU remained constant, all regions, except the South region, showed adherence to the PEC greater than 50% in 2022, demonstrating a significant increase in service use compared with the results obtained in 2017 (Table). As of December 2017, Brazil had 18,510 BHU that used some type of Electronic Health Record (eHR), with only 8,930 opting for the PEC. In December 2022, of the 38,218 BHU that used PE, 26,091 had already joined the PEC^19^.

Therefore, in 2017, the PEC was only the third option among the eHR. Data collected by SISAPS indicate that 52.69% (n = 22598 BHU) of the records were carried out through Simplified Data Collection (CDS), followed by the use of their own systems (22.34%, n = 9,580 BHU). However, by the end of 2022, we can observe a drastic change in the use of eMR by BHU, in which the PEC now represents more than half (59.93%, n = 26,091 BHU) of the eHR used by the Primary Healthcare network (Figure).

**Figure:**
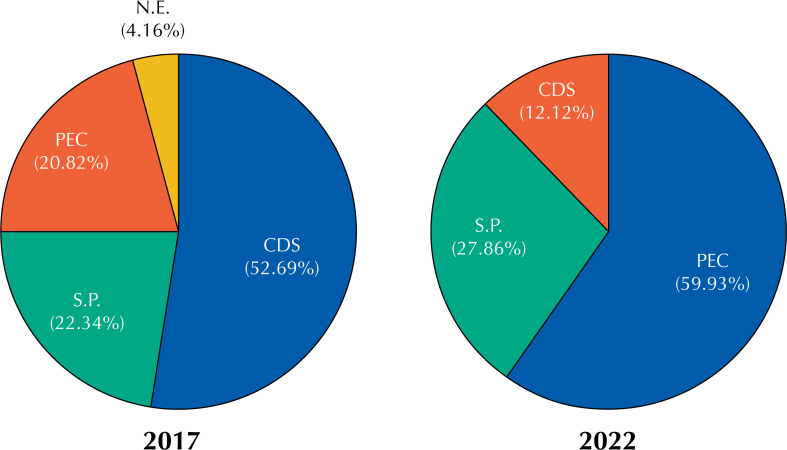
Adherence to the PEC from 2017 to 2022.

Caption: S.P: Own System; N.E: Failure to send information to the Health Information System for Primary Healthcare; SDC: Simplified Data Collection; PEC: Citizen's Electronic Medical Record.

Source: Ministry of Health, 2023 (data collected on 10/17/2023)^19^.

### Ten years of PEC e-SUS APS

#### The genesis of PEC and the system's data structure

In 2011, the Ministry of Health took the initiative to computerize the Brazilian health system within the scope of APS. To this end, a task force was set up years before the launch of the first version of the PEC to build a strategy for implementing an information system to record and manage APS health data.

The working group responsible for developing the e-SUS APS strategy comprised people from the Ministry of Health, representatives from municipal and state health departments, scholars in the field affiliated with Universities, and representatives from the private sector. There were many conversations to exchange experiences and survey the need for using medical records in the APS scenario, where the group had the opportunity to learn about the success story of the city of Florianópolis. This fact influenced the choice of a partner to develop the citizen's electronic Health records. At that moment, the Bridge Laboratory was created, resulting from a partnership between the Ministry of Health and the Federal University of Santa Catarina, which is responsible for developing the PEC e-SUS APS to this day.

The PEC data structure was built based on the Problem-Oriented Medical Registry (POMR), which has three fundamental areas for recording clinical information: the citizen database, the list of problems, and clinical evolution notes structured in the Subjective, Objective, Assessment, and Plan (SOAP) format^20^. In this logic, all system developments were designed and implemented to qualify health records and facilitate the analysis of the patient's condition and clinical history.

The PEC e-SUS APS is available free of charge to all Brazilian municipalities, but it is worth noting that they are not obliged to use the system and can choose to develop their own or hire a private provider. However, regardless of this, the data must follow established criteria for integration and send records to the National Centralizer and, subsequently, to SISAB, which seeks to store health information tactically and individually, aiming for comprehensive monitoring of each user's care^7,21^.

#### Technical evolution of the system

Over the 10 years of the PEC, several system versions have been launched, with technical improvements, new functionalities, and new modules. Brazilian municipalities have gradually implemented these versions based on identifying opportunities for improvement and the need to update and link new functionalities to the policies implemented by the Ministry of Health. Chart 1 summarizes the main versions and functionalities developed, representing the system's technological evolution and adaptation to user needs.

**Chart 1:** Main versions of the PEC e-SUS APS.

Version	Year	Main modules and functionalities released
0.9.17	2013	First production version
1.2	2014	Advances in the Home Care flow Release of the installation wizard New XML/CNES
1.3	2014	Service Attendance Declaration Functionality Release of the Operational Registration, Pregnant Women, Children, and Cardiovascular Risk Reports Restructuring of the Medicines Prescription Review of Exams and Certificates modules New Data Transmission Process
2.0	2015	Release of Food Consumption Markers Release of the Home Care Eligibility Assessment Release of the new Risk Classification model Postgres database support Release of the Pre-Natal Care module Release of the Pre-Natal Monitoring module Release of External Referral Release of the Procedure Service module
2.1	2016	Functionality for requesting justification for access to the Medical Record Release of integration documentation Citizen Appointment List Functionality Improvements to adapt to the SBIS Manual Release of the Zika Virus Notification Sheet Inclusion of Integrative and Complementary Practices in the system (Rationality in Health) Shared Service registration functionality Release of Childcare Service Child Monitoring Module Release of the Family Nucleus Inconsistencies Report Inclusion of the system's Terms of Use Support for Oracle, Postgres, and a customized version of Postgres databases
2.2	2017	Release of the Allergies module Release of new Production Reports Cover Sheet Review
3.0	2017	Release of the Vaccination Service module Release of the Online Agenda module
3.1	2018	Release of the Vaccination Tracking module
3.2	2019	Registration Unification Functionality Release of the Historical Series Reports Previous Vaccination Record Functionality Start of the Support channel/team for municipalities
4.0	2020	Start of system redesign with the Administration Module and general system components
4.1	2020	Change in system name to e-SUS APS Redesign of the Service List Release of the User Satisfaction Survey functionality Health Conditions Monitoring Module
4.2	2020	Release of the Absenteeism Report Redesign of the Home Care module
4.3	2021	Release of the Vaccination Management Report Release of the Service Management Report
4.5	2021	Redesign of the Agenda module Redesign of the Vaccination Service module
5.0	2022	Redesign of the Individual Service module Suitability for use of the PEC in the DSC Redesign of the Odontogram
5.1	2022	Release of the Active Vaccination Search module Functionality of PEC use for Interns Improvements to the Food Consumption Markers module Release of the Reterritorialization module
5.2	2023	Release of the e-SUS APS Video Call module Release of the Electronic Prescription module Sending vaccination data to the RNDS

Caption: DSC: Dental Specialty Center; CNES: *Cadastro Nacional de Estabelecimentos de Saúde* (National Registry of Health Establishments); PEC: Citizen's Electronic Medical Record; RNDS: *Rede Nacional de Dados em Saúde* (National Health Data Network); SBIS: *Sociedade Brasileira de Informática em Saúde* (Brazilian Society of Health Informatics); XML: Extensible Markup Language.

Source: Prepared by the authors (2023).

The first version of PEC e-SUS APS to be made available by municipalities was 0.9.17, which had a reduced scope and few features compared to current system versions. It consisted of a module for managing healthcare units, managing healthcare professionals, registering citizens, attendance lists, individual care, and sending the Outpatient Production Bulletin (OPB), among others. Sending the OPB was an essential functionality for monitoring and evaluating the productivity of BHU in Brazilian municipalities. The data submission models for health policy analysis by the Ministry of Health have changed over the last 10 years, and the PEC e-SUS APS played an essential role in distributing information to government databases. Currently, data is extracted from SISAB following the guidelines of the Programa Previne Brasil^22^.

Over the years, the PEC architecture has also undergone significant transformations, especially concerning the database used. Initially, in versions 0.9.17 and 1.0, H2 was chosen. However, the need for a more robust and scalable database arose, resulting in the adoption of PostgreSQL in version 2.0. Starting with version 2.1, it became possible to choose between three database options, namely Oracle, Postgres, and a customizable version of Postgres. These changes allowed greater installation flexibility to meet each municipality's specific needs.

Among the improvements implemented in these 10 years, those of an administrative nature and management of care and APS services stand out, such as the online agenda functionality (version 3.0) which, integrated with *Conecte SUS*, allows one to schedule appointments remotely at the user's reference BHU^23^; the registration unification functionality (version 3.2), which allows the unification of several registrations of the same citizen, contributing to the cleaning of the database and centralization of records in a single health record; sending vaccination and individual care data to the *Rede Nacional de Dados em Saúde* (RNDS– National Health Data Network) (version 5.2), which contributes to data interoperability in the health system; and the active search module (version 5.1), which allows the issuance of a list of citizens with their appropriate addresses and contacts, facilitating the work ofcommunity health agentsin dialogue with them.

Among the main clinical and care functionalities, the following stand out: the pre-natal care module (version 2.0.0), which enables specific information fields for gestational consultation in APSand qualifies its registration in the system; childcare services (version 2.1), which allows structured recording of assessments of child growth and development and assists in child monitoring practices; the odontogram, which was improved in version 5.0.0 and consisted of a graphic representation of the oral cavity, allowing easier recording of procedures carried out in the dental consultation and qualifying the record of dental care in APS; and the e-SUS PHC video call (version 5.2), which allows video calls between healthcare professionals and patients in an integrated manner with the citizen's medical record, expanding access to healthcare services and specialties.

In recent years, the PEC e-SUS APS has undergone a redesign of its modules, which began with version 4.0 and ended with 5.0. This aimed to improve system usability and user experience. To this end, studies were developed with health professionals from partner municipalities to implement the new versions of the PEC, aiming to understand users’ needs. Notably, all development stages were based on a process/model called b_thinking, a methodology developed by Bridge Laboratory that combines the most recent guidelines on agile development, User Experience (UX), User Interface (UI), and privacy by design.

The versions scheduled to be launched in 2023 mark a new era for the PEC e-SUS APS, bringing advances in new digital health modalities and technologies, such as video calling and digital prescription. The system incorporated a video call module to encourage sharing of care between the APSteam and specialist professionals who provide support through a specialized application for carrying out telehealth practice.

Meanwhile, the digital prescription sought to implement a tool for issuing digitally signed prescriptions, with a mechanism for reading and recognizing the prescription by public medication dispensing services. Issuing prescriptions in this format is an initial step toward digitizing, integrating, and sharing health documents in the public sector.

Since March 2020, 118 system versions have been released with improvements, corrections, and new functionalities, resulting in an average release of three new versions per month. This fact highlights the speed of implementation of new developments and the incentives for the PEC to be developed as a priority electronic health record for use in Brazilian APS according to the guidelines and policies of the Ministry of Health.

#### Applications to support PEC e-SUS APS

During the 10 years of PEC e-SUS APS, several applications were released to be used with health records (Chart 2). Their main contributions are the possibility of offline use in remote environments/external to the BHU and sending forms/records to the National Data Centralizer, reducing rework when typing this information later.

**Chart 2:** Applications of the e-SUS AB strategy.

Release date	Application	Objectives/functionalities
December 2014	*e-SUS Atenção* *Domiciliar*	Its purpose is to record clinical information that will be integrated into the PEC and capture production data for eMAD and eMAP. Its objective is to assist the teams’ work process, seeking to qualify the recording of information, by offering a mobile and electronic tool so that the recording of information takes place at the service location, enabling it to be carried out more quickly, with less risk of data loss.
October 2016	*e-SUS Território*	Used by CHAs, EDCAs, Social Action Agents, and Health Visitors when filling out forms for data collection and, in addition, allowing healthcare units greater knowledge about the territory under their responsibility.
December 2018	*e-SUS Atividade Coletiva*	Used to record group activities, such as meetings, orientation meetings, and health education, among others. The application was structured to facilitate the recording of this information, compared to the recording carried out by PEC/SDC Forms.
March 2022	*e-SUS Vacinação*	Used to record the application of immunobiologicals during vaccination campaigns/actions inside or outside the healthcare unit environment.
June 2022	*Gestão e-SUS APS*	Provides simplified and dynamic access to data from the PEC e-SUS APS Production Reports, and thus favors the use of data during public management processes, and can be used during team meetings with municipal managers and healthcare professionals, among others.

Caption: EDCA: Endemic Disease Control Agent; CHA: Community Health Agent; APS: *Atenção Primaria a Saúde* (Primary Healthcare); SDC: Simplified Data Collection; eMAD: *Equipe Multiprofissionais de Atenção Domiciliar* (Multidisciplinary Home Care Teams); eMAP: *Equipes Multiprofissionais de Apoio* (Multidisciplinary Support Teams); PEC: *Prontuário Eletrônico do Cidadão* (Citizen's Electronic Medical Record).

Source: Prepared by the authors (2023).

#### Difficulties in implementing the PEC in Brazilian municipalities

Studies that analyze the use of the PEC highlight difficulties in its implementation and adherence associated with changes in workflow, professional turnover, and the lack of a team training plan. Added to this are individual obstacles related to skills in using computer equipment, which impact resistance to adherence and the use of electronic health records. Even today, these issues are still a concern, as misuse of the system can directly interfere with the quality of health records^24–26^.

Even though the Ministry of Health has invested in strategies to encourage the computerization of healthcare units and expand the technological capacity of municipalities, the lack of connectivity and low infrastructure have also presented themselves as challenges for implementing the PEC over these 10 years. These problems included structural issues and issues related to the space available for installing equipment, such as computers and network servers. The availability of internet access is still an obstacle, especially in remote and difficult-to-access locations. It is worth remembering that Brazil has continental dimensions and, even today, some places still do not have the infrastructure for the PEC to be implemented^16,27,28^.

Another aspect that may interfere with using PEC over third-party systems is its integration with other health network services, especially at specialized and hospital care levels. In the meantime, it is understood that municipal managers tend to prioritize devices operating at different healthcare points to integrate information at the municipal level.

With the establishment of Previne Brasil, municipalities began to hire the services of private companies to extract data and generate performance indicators for health teams. It is worth noting that the PEC e-SUS APS was not developed to be integrated with third-party systems, whether in data extraction or information sharing between services. Therefore, the importance of holding municipal management and contracted companies responsible for maintaining and adapting systems concerning interoperability, data protection, and security is highlighted.

#### Improvements and paths to be explored

The PEC e-SUS APS has constantly evolved during its operation, and manyfunctionalitieshave already been implemented since its first version. However, there is still much to be done so that citizens, healthcare professionals, and managers benefit from a digital flow of care, recording health information, and strengthening a database for health decision-making. The literature points out that small municipalities’ non-adherence to the PEC e-SUS APS requires the development of specific strategies to promote institutional support in implementing technologies and tackling geographic barriers^10^.

The design of studies and improvements related to usability and user experience must continue to be priority topics in the PEC e-SUS APS development agenda. It is already known that the misuse of electronic medical records can affect the quality of care and patient safety, as inadequate design and functionality can cause fatigue, delays in data recording, and errors/failures in care^29^.

Furthermore, expanding the PEC to other services in the healthcare network can contribute to the longitudinal feature and continuity of care provided to citizens via healthcare professionals’ access to patients’ clinical data in different healthcare services. It is understood that using an integrated medical record in the healthcare network tends to reduce fragmentation in the care delivery system, improving quality and efficiency by reducing gaps in care^30^. From this perspective, it is also expected that the PEC can continue to be a reference in health data interoperability initiatives and solutions by expanding the data shared with the RNDS.

Another suggestion for improvement is related to training on using the system, especially during implementation and significant version updates. Studies highlight that factors such as training and the institution of policies, procedures, and financial incentives can be strategies used to influence the attitudes of healthcare professionals favorably regarding the use of electronic medical records in care^30,31^.

The reports generated by electronic medical records are vital for public management. Based on these reports, investment strategies are defined in campaigns to prevent and promote the health of citizens^32^. The reports generated by the PEC can also be the focus of future improvements, seeking to offer the construction of indicators so that managers can better manage their public resources within the scope of APS.

This study has limitations in not directly approaching system users (healthcare professionals) with interviews, which could have broadened the research analysis perspective. Even so, it is an innovative study, presenting the evolution of medical records and the e-SUS APS strategy from the perspective of the development of functionalities and adherence by municipalities in Brazil.

## FINAL CONSIDERATIONS

In these 10 years of history, it was possible to observe the growth and dissemination of the PEC e-SUS APS among Brazilian municipalities, as shown by data on adherence to the system, which increased exponentially over the years. Even without mandatory installation and use, the PEC became Brazil's leading choice for electronic health records, becoming a reference inside and outside the country. It is understood that the increase in adherence is linked to the constant improvements implemented, scaling the functionalities of the health record and contributing to the creation of several applications linked to the e-SUS APS strategy.

However, many challenges will still be faced for the PEC to operate with full performance and coverage in the SUS. The computerization of healthcare services and the expansion of the system for use in other services in the care network will contribute to the integration and access of clinical records, optimizing resources and continuity of care. This scenario appears to be an objective to be achieved by the country's digital healthcare policies and initiatives, emphasizing integration with the RNDS.

Training teams to use the system also requires greater attention, given the frequent improvements and new versions that are developed annually. In this scenario, the importance of institutional support for municipalities throughout the implementation and use process stands out, intending to consolidate a communication channel for training and assistance and proposing improvements in partnership with the system's end users.
