# Supplementary material for: Ten years of the Citizen's Electronic Health Record e-SUS Primary Healthcare: in search of an electronic Unified Health System
Source: Rev Saude Publica. 2024 Jun 6;58:23. doi: 10.11606/s1518-8787.2024058005770 (PMC11196093; doi:10.11606/s1518-8787.2024058005770)
Supplement: Translation

# Translation: Dez anos do Prontuário Eletrônico do Cidadão e-SUS APS: em busca de um Sistema Único de Saúde eletrônico

**Authors:** Ianka Cristina Celuppi, Eduarda Talita Bramorski Mohr, Mariano Felisberto, Thiago Serafim Rodrigues, Jades Fernando Hammes, Célio Luiz Cunha, Raul Sidnei Wazlawick, Eduardo Monguilhott Dalmarco

**Affiliations:** I Florianópolis SC Brasil Universidade Federal de Santa Catarina. Centro Tecnológico. Laboratório Bridge. Florianópolis, SC, Brasil; II Florianópolis SC Brasil Universidade Federal de Santa Catarina. Centro de Ciências da Saúde. Programa de Pós-Graduação em Enfermagem. Florianópolis, SC, Brasil; III Florianópolis SC Brasil Universidade Federal de Santa Catarina. Centro de Ciências da Saúde. Programa de Pós-Graduação em Farmácia. Florianópolis, SC, Brasil; IV Florianópolis SC Brasil Universidade Federal de Santa Catarina. Centro Tecnológico. Departamento de Ciências da Computação. Florianópolis, SC, Brasil

**Keywords:** Registros Eletrônicos de Saúde, Tecnologia Digital, Atenção Primária à Saúde, Política de Saúde, Sistema Único de Saúde

## Abstract

**OBJETIVO:**

Contextualizar a adesão ao Prontuário Eletrônico do Cidadão (PEC) pelos municípios brasileiros e a evolução da estratégia eletrônica do Sistema Único de Saúde (e-SUS) da Atenção Primária (APS) durante seus 10 anos.

**MÉTODOS:**

Trata-se de um estudo de cunho descritivo, que agregou informações de adesão ao uso do prontuário, extraídas da base de dados da Secretaria de Atenção Primária à Saúde (SAPS) do Governo Federal, entre os anos de 2017 e 2022. Foram analisados o quantitativo de unidades básicas de saúde informatizadas que utilizavam algum prontuário eletrônico e o número das que utilizavam a Coleta de Dados Simplificada (CDS) e das que implementaram o Prontuário Eletrônico do Cidadão (PEC), no mesmo período. Também foi realizada uma síntese descritiva das funcionalidades e módulos que foram implementados no sistema durante seus 10 anos de desenvolvimento.

**RESULTADOS:**

A adesão dos municípios brasileiros ao PEC cresceu exponencialmente nos últimos cinco anos, passando de 8.930 unidades de saúde em 2017 para 26.091 em 2022. Como era de se esperar, as principais funcionalidades e melhorias desenvolvidas nessa década buscaram implementar novos fluxos e módulos de processos administrativos, de atendimento clínico e de gestão do cuidado e administração do serviço de saúde, mas também foram importantes para o sucesso do sistema aprimorar aspectos de usabilidade e de infraestrutura tecnológica da arquitetura da aplicação.

**CONCLUSÕES:**

Em 2023, celebra-se o marco de uma década do início da implantação do prontuário pelos municípios brasileiros, marcado por desafios de ordem tecnológica e de infraestrutura, bem como de melhorias e novas funcionalidades que evidenciaram a evolução tecnológica do sistema e da estratégia e-SUS APS. Mesmo que muitas outras existam, pode-se dizer que o PEC é hoje a principal ferramenta de prontuário eletrônico no Brasil, pois sempre investiu em evolução, vindo a se atualizar nas oportunidades tecnológicas e de usabilidade.

## INTRODUÇÃO

A implementação do Sistema Único de Saúde (SUS) em 1988 viabilizou a criação de uma tríade de princípios baseados na universalidade, integralidade e equidade dos serviços de saúde. Em 1990, a Atenção Primária à Saúde (APS) se firmou como política nacional, a partir da Norma Operacional Básica 96 (NOB 96), que garantiu fomentos para a implementação dos programas de Saúde da Família e dos Agentes Comunitários de Saúde (ACS) em todo o território brasileiro^1,2^.

Nessa perspectiva, a APS desempenha um papel central dentro da organização da rede de atenção à saúde, sendo considerada a principal porta de acesso da população ao sistema de saúde brasileiro e ampliando a oferta de cuidado em saúde no território. Atualmente, segundo a Política Nacional da Atenção Básica (PNAB) de 2017, a APS deve englobar práticas de cuidado integrado e gestão qualificada, prezando pelo desempenho multiprofissional em território definido^3–5^.

Os cuidados prestados na APS reduziram significativamente os números de internações hospitalares ao longo dos anos, diminuindo os gargalos formados principalmente por pacientes com complicações advindas de doenças crônicas, como hipertensão e diabetes^6–8^. Entretanto, alguns desafios ainda precisam ser superados pela gestão pública, como a recente diminuição da cobertura vacinal, a ampliação do acesso a esse serviço, a melhoria da qualidade do cuidado às condições crônicas, a redução da transmissão de doenças infecciosas, dentre outros aspectos^7,9^.

Nos últimos 10 anos, com o advento da tecnologia e a construção de ecossistemas digitais no setor da saúde, foram incorporadas mudanças na APS relacionadas à informatização dos serviços e à adesão ao uso de tecnologias. A Estratégia e-SUS Atenção Básica (e-SUS AB) foi criada a partir da reestruturação do Sistema de Informação em Saúde para a Atenção Básica (SISAB) em 2013 e tem como finalidade ampliar, reestruturar e garantir uma interação efetiva dos sistemas de informação que permeiam a saúde no Brasil^10^. Com isso, a estratégia e-SUS AB, atualmente chamada de estratégia e-SUS APS, busca expandir a informatização no SUS, contribuindo para a qualificação dos registros, a integração de informações e a otimização da análise de dados em saúde, mediante a introdução de novas tecnologias^10,11^.

Nessa lógica, o Ministério da Saúde disponibilizou para uso gratuito dos municípios o Prontuário Eletrônico do Cidadão (PEC), que busca facilitar o processo de informatização das unidades básicas de saúde (UBS) em todo o território brasileiro. Durante os 10 anos de implementação do prontuário, também foram desenvolvidos o Centralizador Nacional de dados e diversos aplicativos *mobile* de apoio aos profissionais, como o e-SUS Atenção Domiciliar, o e-SUS Atividade Coletiva, o e-SUS Território, o e-SUS Vacinação e o Gestão e-SUS APS^12^. A estratégia e-SUS APS também disponibiliza o sistema de Coleta de Dados Simplificado (CDS), considerado uma ferramenta de transição ao modelo informatizado, no qual são coletados dados por meio de fichas com posterior digitalização, permitindo que sejam utilizados em cenários de baixa infraestrutura tecnológica e conectividade^11,13^.

O processo de implementação do PEC e-SUS APS pode ser influenciado pelas características dos municípios, como sua localização, densidade populacional, urbanização, preparo e assessoria da gestão municipal, nível de informatização e infraestrutura tecnológica, entre outras^10^. Tais aspectos, quando associados a outros desafios enfrentados pelos profissionais de saúde e pelos gestores, podem determinar a adesão da região ao projeto. A literatura destaca algumas dificuldades de implementação e uso do sistema, como a insuficiência de recursos materiais nos municípios, o déficit de treinamento dos profissionais para a utilização da tecnologia e a baixa conectividade com a internet^14–16^.

Nesse ínterim, a implementação do PEC e-SUS APS e demais aplicativos voltados à área da saúde representam mudanças estruturais nas tecnologias e nos processos de trabalho já consolidados no Brasil, levando à informatização e ao fortalecimento da saúde digital no país. Tal fato justifica a relevância do desenvolvimento deste estudo, que sintetiza a evolução tecnológica de uma política governamental de sucesso e de grande impacto na APS brasileira, que completa uma década de implementação no ano de 2023. Diante disso, o objetivo deste trabalho é contextualizar a adesão ao Prontuário Eletrônico do Cidadão pelos municípios brasileiros e a evolução da estratégia e-SUS APS durante seus 10 anos de existência.

## MÉTODOS

Trata-se de um estudo descritivo, que agregou informações de adesão ao uso do PEC, extraídos da base de dados da Secretaria de Atenção Primária à Saúde (SAPS), entre os anos de 2017 e 2022. Também foi realizada uma síntese descritiva das funcionalidades e módulos que foram implementados no sistema durante seus 10 anos de evolução, apresentando um panorama da sua consolidação tecnológica.

### Adesão dos municípios brasileiros ao PEC e-SUS APS

Ao longo dos anos, podemos observar o crescimento da consolidação do PEC em todo o território brasileiro, juntamente com o aumento e a modernização das UBS. Esse crescimento demonstra um esforço conjunto para unificar e desfragmentar as informações e serviços prestados na atenção primária à saúde^17,18^. Segundo informações coletadas da base de dados da SAPS, entre 2017 (ano que iniciou a verificação por parte da SAPS) e 2022 houve um aumento exponencial da implementação do PEC em todas as regiões brasileiras, destacando-se o Nordeste (367,72%), o Norte (256,10%) e o Sudeste (157,04%) (Tabela).

**Tabela:** Unidades básicas de saúde (UBS) com implementação do Prontuário Eletrônico do Cidadão (PEC) entre os anos de 2017 a 2022:

Ano	Regiões do Brasil									
	Norte		Nordeste		Centro-Oeste		Sudeste		Sul	
	UBS^a^	PEC^b^	UBS^a^	PEC^b^	UBS^a^	PEC^b^	UBS^a^	PEC^b^	UBS^a^	PEC^b^
2017	751	631 (84,02%)	3.955	2.618 (66,19%)	2.068	1.014 (49,03%)	6.411	2.724 (42,48%)	5.325	1.943 (36,48%)
2018	1.008	841 (83,43%)	4.763	3.232 (67,85%)	2.252	1.255 (55,72%)	7.411	3.273 (44,16%)	5.707	1.997 (34,99%)
2019	1.320	1.099 (83,25%)	6.911	5.018 (72,60%)	2.524	1.618 (64,10%)	9.057	4.708 (51,98%)	6.096	2.354 (38,61%)
2020	1.829	1.553 (84,90%)	9.919	7.905 (79,69%)	2.782	1.891 (67,97%)	11.026	6.364 (57,71%)	6.322	2.557 (40,44%)
2021	2.117	1.802 (85,12%)	11.772	9.843 (83,61%)	2.923	2.090 (71,50%)	11.630	6.705 (57,65%)	6.468	2.495 (38,57%)
2022	2.531	2.247 (88,77%)	13.942	12.245 (87,82%)	2.977	2.201 (73,93%)	12.284	7.002 (57,00%)	6.484	2.396 (36,95%)

Implementação e utilização do PEC no Brasil ao longo de 6 anos (2017-2022), onde ^a^representa o número de UBS em funcionamento que utilizavam algum PE em dezembro de cada ano e ^b^representa o número de UBS que optaram pela implementação e utilização do PEC em dezembro de cada ano. A porcentagem (%) representa o número de UBS com PEC^b^ em relação ao total de UBS^a^. Ministério da Saúde, 2023 (dados coletados em 17/10/2023)^19^.

Embora o número total de UBS tenha se mantido constante, todas as regiões, com exceção da região Sul, apresentaram uma adesão ao PEC superior a 50% em 2022, demonstrando um aumento significativo na utilização do serviço quando comparado aos resultados obtidos em 2017 (Tabela). Até dezembro de 2017, o Brasil possuía 18.510 UBS que utilizavam algum tipo de Prontuário Eletrônico (PE), sendo que apenas 8.930 optaram pelo PEC. Já em dezembro de 2022, das 38.218 UBS que utilizavam PE, 26.091 já haviam aderido ao PEC^19^.

Sendo assim, em 2017, o PEC era apenas a terceira opção dentre os PE. Dados coletados pelo SISAPS apontam que 52,69% (n = 22.598 UBS) dos registros foram realizados através da Coleta de Dados Simplificados (CDS), seguido pela utilização de Sistemas Próprios (22,34%, n = 9.580 UBS). Entretanto, até o final de 2022, podemos observar uma mudança drástica na utilização de PE pelas UBS, em que o PEC passa a representar mais da metade (59,93%, n = 26.091 UBS) dos PE utilizados pela rede de Atenção Básica (Figura).

**Figura:** Adesão ao PEC entre os anos de 2017 e 2022.

Legenda: S.P: Sistema Próprio; N.E: Não envio de informação ao Sistema de Informação em Saúde para a Atenção Básica; CDS: Coleta de Dados Simplificada; PEC: Prontuário Eletrônico do Cidadão.

Fonte: Ministério da Saúde, 2023 (dados coletados em 17/10/2023) ^19^.

### Dez anos de PEC e-SUS APS

#### A gênese do PEC e a estrutura de dados do sistema

Em 2011, partiu do Ministério da Saúde a iniciativa de informatizar o sistema de saúde brasileiro no âmbito da APS. Para isso, foi montada uma força tarefa, anos antes do lançamento da primeira versão do PEC, para construir uma estratégia de implementação de um sistema de informação, visando o registro e a gestão dos dados de saúde da APS.

O grupo de trabalho que ficou responsável pelo desenvolvimento da estratégia e-SUS APS era formado por pessoas do Ministério da Saúde, representantes das secretarias municipais e estaduais de saúde, estudiosos da área filiados a universidades e representantes da iniciativa privada. Houve muitas conversas para troca de experiências e levantamento das necessidades de uso de um prontuário no cenário da APS, onde o grupo teve a oportunidade de conhecer o caso de sucesso da cidade de Florianópolis. Este fato influenciou na escolha de um parceiro para ser desenvolvedor do prontuário eletrônico do cidadão. Nesse momento nasceu o Laboratório Bridge, fruto da parceria entre o Ministério da Saúde e a Universidade Federal de Santa Catarina, que é responsável pelo desenvolvimento do PEC e-SUS APS até hoje.

A estrutura de dados do PEC foi construída com base no Registro Clínico Orientado à Problemas (RCOP), que possui três áreas fundamentais para registro das informações clínicas: a base de dados do cidadão; a lista de problemas; e as notas de evolução clínica, estruturadas no formato Subjetivo, Objetivo, Avaliação e Plano (SOAP)^20^. Nessa lógica, todas as evoluções do sistema foram pensadas e implementadas para qualificar os registros em saúde e favorecer a análise da condição e histórico clínico do paciente.

O PEC e-SUS APS é disponibilizado gratuitamente para todos os municípios brasileiros, mas cabe ressaltar que estes não são obrigados a utilizar o sistema, podendo optar por desenvolver seus próprios ou contratar um privado. Porém, independentemente disso, os dados devem seguir critérios estabelecidos para integração e envio dos registros ao Centralizador Nacional e, posteriormente, ao SISAB, que busca armazenar as informações em saúde taticamente e de forma individualizada, objetivando um acompanhamento integral dos atendimentos de cada usuário^7,21^.

#### Evolução técnica do sistema

Ao longo dos 10 anos do PEC, foram lançadas diversas versões do sistema, com melhorias técnicas, novas funcionalidades e novos módulos. Essas versões vêm sendo implementadas de forma gradual pelos municípios brasileiros, a partir da identificação de oportunidades de melhoria e de necessidades de atualização e vinculação de novas funcionalidades às políticas executadas pelo Ministério da Saúde. O Quadro 1 sintetiza as principais versões e funcionalidades desenvolvidas, que representam a evolução do sistema na perspectiva tecnológica e de adequação às necessidades dos usuários.

**Quadro 1:** Principais versões do PEC e-SUS APS.

Versão	Ano	Principais módulos e funcionalidades lançadas
0.9.17	2013	Primeira versão de produção
1.2	2014	Avanços no fluxo de Atenção Domiciliar Lançamento do wizard de instalação Novo XML/CNES
1.3	2014	Funcionalidade de Declaração de Comparecimento ao Atendimento Lançamento dos Relatórios Operacionais de Cadastro, Gestantes, Crianças e Risco Cardiovascular Reestruturação do Receituário de Medicamentos Revisão dos módulos de Exames e Atestados Novo Processo de Transmissão de Dados
2.0	2015	Lançamento dos Marcadores de Consumo Alimentar Lançamento da Avaliação de Elegibilidade para Atenção Domiciliar Lançamento do novo modelo de Classificação de Risco Suporte para o banco de dados Postgres Lançamento do módulo de Atendimento de Pré-Natal Lançamento do módulo de Acompanhamento de Pré-Natal Lançamento de Encaminhamento Externo Lançamento do módulo de Atendimento de Procedimentos
2.1	2016	Funcionalidade de solicitação de justificativa para acesso ao Prontuário Lançamento da documentação de integração Funcionalidade de Lista de Agendamentos do Cidadão Melhorias para adequação ao Manual da SBIS Lançamento da Ficha de Notificação do Zika Vírus Inclusão das Práticas Integrativas e Complementares no sistema (Racionalidade em Saúde) Funcionalidade de registro de Atendimento Compartilhado Lançamento do Atendimento de Puericultura Módulo de Acompanhamento da Criança Lançamento do Relatório de Inconsistências do Núcleo Familiar Inclusão dos Termos de Uso do sistema Suporte aos bancos Oracle, Postgres e uma versão personalizada do Postgres
2.2	2017	Lançamento do módulo de Alergias Lançamento de novos Relatórios de Produção Revisão da Folha de Rosto
3.0	2017	Lançamento do módulo de Atendimento de Vacinação Lançamento do módulo de Agenda Online
3.1	2018	Lançamento do módulo de Acompanhamento de Vacinação
3.2	2019	Funcionalidade de Unificação de Cadastros Lançamento dos Relatórios de Série Histórica Funcionalidade de Registro Anterior de Vacinação Início do canal/equipe de Suporte aos municípios
4.0	2020	Início do redesign do sistema com o Módulo de Administração e componentes gerais do sistema
4.1	2020	Alteração no nome do sistema para e-SUS APS Redesign da Lista de Atendimentos Lançamento da funcionalidade de Pesquisa de Satisfação dos usuários Módulo de Acompanhamento de Condições de Saúde
4.2	2020	Lançamento do Relatório de Absenteísmo Redesign do módulo de Atenção Domiciliar
4.3	2021	Lançamento do Relatório Gerencial de Vacinação Lançamento do Relatório Gerencial de Atendimentos
4.5	2021	Redesign do módulo de Agenda Redesign do módulo de Atendimento de Vacinação
5.0	2022	Redesign do módulo de Atendimento Individual Adequação para uso do PEC no CEO Redesign do Odontograma
5.1	2022	Lançamento do módulo de Busca Ativa de Vacinação Funcionalidade de uso do PEC para Estagiários Melhorias no módulo de Marcadores de Consumo Alimentar Lançamento do módulo de Reterritorialização
5.2	2023	Lançamento do módulo de Vídeo Chamada e-SUS APS Lançamento do módulo de Prescrição Eletrônica Envio dos dados de vacinação para a RNDS

Legenda: CEO: Centro de Especialidades Odontológicas; CNES: Cadastro Nacional de Estabelecimentos de Saúde; PEC: Prontuário Eletrônico do Cidadão; RNDS: Rede Nacional de Dados em Saúde; SBIS: Sociedade Brasileira de Informática em Saúde; XML: Extensible Markup Language.

Fonte: Elaboração dos autores (2023).

A primeira versão do PEC e-SUS APS a ser disponibilizada para uso dos municípios foi a 0.9.17, que apresentava escopo reduzido e poucas funcionalidades se comparado com versões atuais do sistema. Ela era composta por um módulo de gestão das unidades de saúde, gestão dos profissionais de saúde, cadastro de cidadãos, lista de atendimentos, atendimento individual, envio do Boletim de Produção Ambulatorial (BPA), entre outros. O envio do BPA era uma funcionalidade essencial para monitorar e avaliar a produtividade das UBS nos municípios brasileiros. Os modelos de envio de dados para análise da política de saúde pelo MS sofreram alterações durante os últimos 10 anos, e o PEC e-SUS APS desempenhou importante papel na distribuição das informações para os bancos de dados governamentais. Atualmente, os dados são extraídos do SISAB mediante as diretrizes do Programa Previne Brasil^22^.

Ao longo dos anos, a arquitetura do PEC também passou por transformações significativas, especialmente no que se refere ao banco de dados utilizado. Inicialmente, nas versões 0.9.17 e 1.0, optou-se pelo H2. No entanto, surgiu a necessidade de um banco de dados mais robusto e escalável, resultando na adoção do *PostgreSQL* na versão 2.0. A partir da versão 2.1, se tornou possível escolher entre três opções de banco de dados, sendo elas: *Oracle*, *Postgres* e uma versão personalizável do *Postgres*. Essas mudanças permitiram maior flexibilização na instalação, para as atender às necessidades específicas de cada município.

Entre as melhorias implementadas nestes 10 anos, destacam-se as de cunho administrativo e de gestão do cuidado e do serviço de APS, a exemplo da funcionalidade de agenda online (versão 3.0) que, de maneira integrada ao Conecte SUS, permite agendar consultas remotamente na UBS de referência do usuário^23^; da funcionalidade de unificação de cadastros (versão 3.2), que permite unificar diversos cadastros de um mesmo cidadão, contribuindo para a higienização do banco de dados e centralização dos registros em um único prontuário; do envio de dados de vacinação e de atendimento individual para a Rede Nacional de Dados em Saúde (RNDS) (versão 5.2), que contribui para a interoperabilidade dos dados no sistema de saúde; e do módulo de busca ativa (versão 5.1), que permite a emissão de uma lista de cidadãos com seus devidos endereços e contatos, facilitando o trabalho dos Agentes Comunitários de Saúde (ACS) no diálogo com esses.

Entre as principais funcionalidades clínicas e de cuidado, destacam-se: o módulo de atendimento de pré-natal (versão 2.0.0), que habilita campos de informações específicas da consulta gestacional na APS e qualifica o seu registro no sistema; o atendimento de puericultura (versão 2.1), que permite o registro estruturado da avaliação de crescimento e desenvolvimento infantil e auxilia nas práticas de acompanhamento da criança; o odontograma, que foi melhorado na versão 5.0.0 e consiste em uma representação gráfica da cavidade oral, permitindo o registro facilitado dos procedimentos realizados na consulta odontológica e qualificando o registro dos cuidados odontológicos na APS; e a vídeo chamada e-SUS APS (versão 5.2), que permite a realização de chamadas de vídeo entre profissionais de saúde e pacientes de maneira integrada ao prontuário do cidadão, ampliando o acesso aos serviços e às especialidades de saúde.

Nos últimos anos o PEC e-SUS APS passou pelo redesign de seus módulos, que se iniciou na versão 4.0 e finalizou na 5.0. Isso teve como objetivo melhorar a usabilidade do sistema e a experiência do usuário. Para tal, foram desenvolvidos estudos com profissionais de saúde de municípios parceiros na implantação das novas versões do PEC, com o intuito de compreender as necessidades dos usuários. Cabe destacar que todas as etapas de desenvolvimento foram pautadas em um processo/modelo chamado *b_thinking*, uma metodologia desenvolvida pelo laboratório *Bridge* que congrega as mais recentes orientações acerca do desenvolvimento ágil, *User Experience* (UX), *User Interface* (UI) e *privacy by design*.

As versões que tinham previsão de lançamento para o ano de 2023 marcam uma nova era para o PEC e-SUS APS, trazendo avanços com relação às novas modalidades e tecnologias de saúde digital, como a vídeo chamada e a prescrição digital. O sistema incorporou um módulo de vídeo chamada, buscando fomentar o compartilhamento de cuidado entre a equipe de APS e os profissionais especialistas que prestam apoio mediante o uso de uma aplicação especializada para a realização das práticas de telessaúde.

Enquanto isso, a prescrição digital buscou implementar uma ferramenta de emissão de receituários assinados digitalmente, com mecanismo de leitura e reconhecimento da prescrição pelos serviços públicos de dispensação de medicamentos. A emissão de prescrições nesse formato se caracteriza como um passo inicial rumo à digitalização, à integração e ao compartilhamento de documentos de saúde no setor público.

Desde março de 2020, foram lançadas 118 versões do sistema com melhorias, correções e novas funcionalidades, resultando em uma média de lançamento de três novas versões por mês. Tal fato destaca a velocidade de implementação de novidades e os incentivos para que o PEC se desenvolva enquanto prontuário eletrônico prioritário para uso na APS brasileira, segundo as diretrizes e políticas do Ministério da Saúde.

#### Aplicativos de apoio ao PEC e-SUS APS

Durante os 10 anos de PEC e-SUS APS, foram lançados diversos aplicativos para uso conjunto com o prontuário (Quadro 2). As principais contribuições deles são a possibilidade de uso *offline* e em ambientes remotos/externos à UBS, bem como o envio de fichas/registros ao Centralizador Nacional de Dados, diminuindo o retrabalho na digitação dessas informações em momentos posteriores.

**Quadro 2:** Aplicativos da estratégia e-SUS AB.

Data de lançamento	Aplicativo	Objetivos/funcionalidades
Dezembro 2014	*e-SUS Atenção Domiciliar*	Tem como finalidade o registro de informações clínicas que serão integradas ao PEC, além de captar dados de produção para as EMAD e EMAP. Seu objetivo é auxiliar o processo de trabalho das equipes, buscando qualificar o registro de informações, por meio da oferta de uma ferramenta móvel e eletrônica para que o registro de informações ocorra no local de atendimento, possibilitando realizá-lo de forma mais rápida e com menos risco de perda de dados.
Outubro 2016	*e-SUS Território*	Utilizado por ACSs, ACEs, Agentes de Ação Social e Visitadores Sanitários no preenchimento das fichas para coleta de dados e, além disso, permitir às unidades de saúde maior conhecimento sobre o território que é de sua responsabilidade.
Dezembro 2018	*e-SUS Atividade Coletiva*	Utilizado para registrar atividades em grupo, como reuniões, encontros para orientação, educação em saúde, dentre outros. O aplicativo foi estruturado de modo a facilitar o registro dessas informações, se comparado ao registro realizado pelo PEC/Fichas CDS.
Março 2022	*e-SUS Vacinação*	Utilizado para registro de aplicação de imunobiológicos durante campanhas/ações de vacinação dentro ou fora do ambiente da unidade de saúde.
Junho 2022	*Gestão e-SUS APS*	Fornece acesso simplificado e dinâmico aos dados dos Relatórios de Produção do PEC e-SUS APS, e com isso favorece o uso de dados durante os processos de gestão pública, podendo ser utilizado em momentos de reuniões da equipe, com gestores municipais, profissionais de saúde, dentre outros.

Legenda: ACE: Agente de Combate à Endemias; ACS: Agente Comunitário de Saúde; APS: Atenção Primária à Saúde; CDS: Coleta de Dados Simplificada; EMAD: Equipes Multiprofissionais de Atenção Domiciliar; EMAP: Equipes Multiprofissionais de Apoio; PEC: Prontuário Eletrônico do Cidadão.

Fonte: Elaboração dos autores (2023).

#### Dificuldades para implementação do PEC nos municípios brasileiros

Estudos que analisam o uso do PEC destacam dificuldades para sua implementação e adesão, associadas às mudanças de fluxo de trabalho, à rotatividade de profissionais e à falta de um plano de treinamento das equipes. Somado a isso, acrescentam-se os obstáculos individuais relacionados às habilidades no uso de equipamentos de informática, que impactam na resistência para adesão e utilização do prontuário eletrônico. Mesmo hoje, essas questões ainda preocupam, pois o mal uso do sistema pode interferir diretamente na qualidade dos registros em saúde^24–26^.

Por mais que o Ministério da Saúde tenha investido em estratégias de estímulo à informatização das unidades de saúde e em ampliação da capacidade tecnológica dos municípios, a falta de conectividade e baixa infraestrutura também se apresentam como desafios para a implementação do PEC nestes 10 anos. Esses problemas passaram por questões relacionadas ao espaço disponível para instalação dos equipamentos, como computadores e servidores de rede, e também por questões estruturais. A disponibilidade de acesso à internet ainda se configura como um empecilho, principalmente em locais remotos e de difícil acesso. Vale lembrar que o Brasil possui dimensões continentais e, mesmo hoje, alguns locais ainda não dispõem de infraestrutura para que o PEC seja implementado^16,27,28^.

Outro aspecto que pode interferir na escolha pelo uso do PEC em detrimento de sistemas terceiros é a sua integração com outros serviços da rede de saúde, especialmente nos níveis da atenção especializada e hospitalar. Nesse ínterim, entende-se que os gestores municipais tendem a priorizar aparelhos que operem nos diferentes pontos de atenção à saúde, com vistas à integração das informações no âmbito municipal.

Com a instituição do Previne Brasil, os municípios passaram a contratar o serviço de empresas privadas para a extração de dados e geração de indicadores de desempenho das equipes de saúde. Cabe destacar que o PEC e-SUS APS não foi desenvolvido com o objetivo de ser integrado a sistemas terceiros, seja no âmbito de extração de dados ou compartilhamento de informações entre serviços. Dessa forma, ressalta-se a importância da responsabilização da gestão municipal e das empresas contratadas na manutenção e adequação dos sistemas no que concerne à interoperabilidade, a proteção e a segurança dos dados.

#### Melhorias e caminhos a serem explorados

Em seu período de funcionamento, o PEC e-SUS APS evoluiu constantemente e muitas funcionalidades já foram implementadas desde a sua primeira versão. Porém, ainda há muito a ser feito para que os cidadãos, profissionais de saúde e gestores sejam beneficiados com um fluxo digital de atendimento, registro das informações em saúde e fortalecimento de uma base de dados para tomada de decisão em saúde. A literatura aponta que a não adesão ao PEC e-SUS APS por municípios de pequeno porte requer o desenvolvimento de estratégias específicas, para promover o apoio institucional na implementação de tecnologias e o enfrentamento de barreiras geográficas^10^.

A concepção de estudos e melhorias relacionadas à usabilidade e à experiência do usuário devem continuar sendo pautas prioritárias na agenda de desenvolvimento do PEC e-SUS APS. Já se sabe que o mau uso dos prontuários eletrônicos pode afetar a qualidade do atendimento e a segurança do paciente, pois um design e funcionalidades inadequadas podem causar fadiga, atraso no registro de dados e erros/falhas no cuidado^29^.

Além disso, a ampliação do PEC para outros serviços da rede de atenção à saúde pode contribuir com a longitudinalidade e continuidade do cuidado prestado ao cidadão, mediante o acesso dos profissionais de saúde aos dados clínicos do paciente em diferentes serviços de saúde. Entende-se que o uso de um prontuário integrado na rede de saúde tende a diminuir a fragmentação no sistema de prestação de cuidados, melhorando a qualidade e a eficiência ao reduzir as lacunas nos cuidados ^30^. Nessa perspectiva, também se espera que o PEC possa continuar sendo referência nas iniciativas e nas soluções de interoperabilidade dos dados em saúde, mediante a ampliação do conjunto de dados compartilhados com a RNDS.

Outra sugestão de melhoria está relacionada aos treinamentos para uso do sistema, especialmente no momento de implementação e nas grandes atualizações de versões. Estudos destacam que fatores como o treinamento e a instituição de políticas, procedimentos e incentivos financeiros podem ser estratégias usadas para influenciar favoravelmente as atitudes dos profissionais de saúde com relação ao uso de prontuários eletrônicos no cuidado^30,31^.

Os relatórios gerados por prontuários eletrônicos são de suma importância para a gestão pública. Com base nesses relatórios, são definidas estratégias de investimento em campanhas de prevenção e promoção da saúde dos cidadãos^32^. Os relatórios gerados pelo PEC também podem ser foco de melhorias futuras, buscando oferecer a construção de indicadores para que os gestores possam administrar melhor os seus recursos públicos no âmbito da APS.

Este estudo apresenta limitações ao não abordar diretamente os usuários do sistema (profissionais de saúde) por meio de entrevistas, o que poderia ter ampliado a perspectiva de análise da pesquisa. Ainda assim, se configura como um estudo inovador, ao apresentara evolução do prontuário e da estratégia e-SUS APS na perspectiva do desenvolvimento de funcionalidades e da adesão pelos municípios do Brasil.

## CONSIDERAÇÕES FINAIS

Nesses 10 anos de história, foi possível observar o crescimento e a disseminação do PEC e-SUS APS entre os municípios brasileiros, como mostram os dados de adesão ao sistema, que aumentaram exponencialmente ao longo dos anos. Mesmo sem a obrigatoriedade de instalação e uso, o PEC passou a ser a principal escolha de prontuário eletrônico no Brasil, tornando-se referência dentro e fora do país. Entende-se que o aumento da adesão está atrelado às constantes melhorias implementadas, escalonando as funcionalidades do prontuário e contribuindo para a criação de diversos aplicativos vinculados a estratégia e-SUS APS.

Entretanto, muitos desafios ainda precisarão ser enfrentados para que o PEC opere com total desempenho e abrangência no SUS. A informatização dos serviços de saúde e a ampliação do sistema para uso em outros serviços da rede de atenção irão contribuir para a integração e acesso dos registros clínicos, otimizando os recursos e a continuidade do cuidado. Tal cenário se configura como um objetivo a ser alcançado pelas políticas e iniciativas de saúde digital do país, com ênfase para a integração com a RNDS.

O treinamento das equipes para utilização do sistema também carece de maior atenção, visto as frequentes melhorias e novas versões que são desenvolvidas anualmente. Nesse cenário, destaca-se a importância do apoio institucional aos municípios durante todo o processo de implantação e uso, com vistas a consolidar um canal de comunicação para treinamentos, auxílio e proposição de melhorias em parceria com os usuários finais do sistema.
